# Snakes and Ladders: A technological approach to tool maintenance byproducts using module flake categories

**DOI:** 10.1007/s10816-025-09754-0

**Published:** 2025-11-24

**Authors:** David Nora, Ariel Malinsky-Buller, Boris Gasparyan, Artur Petrosyan, Ellery Frahm

**Affiliations:** 1 Human-Environment Dynamics Laboratory (HUMENDY), Institute of Archaeology, The Hebrew University of Jerusalem, Mt. Scopus, 91905 Jerusalem, Israel; 2 ICArEHB, Interdisciplinary Center for Archaeology and Evolution Human Behaviour, Universidade Do Algarve, Campus de Gambelas, 8005-139 Faro, Portugal; 3 Institute of Archaeology and Ethnography, National Academy of Sciences of the Replubic of Armenia, Yerevan, Armenia; 4 Department of Anthropology & Council On Archaeological Studies, Yale University, New Haven, CT USA; 5 Anthropology Division, Yale Peabody Museum, New Haven, CT USA

**Keywords:** Lithic technology, Tool maintenance, Curation, Microdebitage, Module flakes, Technological organisation

## Abstract

The study of retouching, reshaping, and rejuvenation in lithic technology has traditionally focused on finished tools, overlooking the byproducts of these processes, particularly microdebitage. This emphasis has led to an incomplete understanding of the dynamic behaviours associated with tool maintenance and a lack of crucial information about prehistoric technological strategies. In this study, we address this knowledge gap. Specifically, we introduce a classification system for lithic byproducts resulting from retouching, reshaping, and rejuvenation techniques, categorising them into five modules (M0 through M4) based on lithic technological analysis. This methodology integrates the *chaîne opératoire* approach to analyse flakes without size thresholds. To demonstrate our approach, we apply it to lithic assemblages from two Middle Palaeolithic sites in Armenia, Kalavan 2 and Ararat-1 Cave. This enables a precise reconstruction of tool use-life and, in turn, the maintenance strategies of Pleistocene hunter-gatherers. Our findings demonstrate that microdebitage (byproducts) can contribute to a holistic view of decision-making, revealing patterns in tool maintenance and raw material provisioning. The module system provides insights on ‘ghost tools’ i., e., tools that are no longer present in the archaeological record, as well as curation behaviours and economic decisions regarding raw materials that were previously difficult to discern. By shifting the focus from finished artefacts to byproducts, this framework enhances our ability to interpret lithic assemblages and understand the adaptive strategies of prehistoric hunter-gatherers.

## Introduction

The study of retouched lithic pieces has played a key role in the systematics of Palaeolithic research since its earliest stages (Monnier, 2006; Trigger, 2006; Wargo, 2009). A well-known example is Bordes’ nomenclature for Lower and Middle Palaeolithic artefacts, which created a common language that remains foundational in the classification of lithic objects into types (Bordes, 1953, 1951, 1969, 1961). Bordes’ system focused on morphology, i.e., the form of the object and the location of retouch as diagnostic components. Similarly, (Brézillon, 1968) defined within a typological approach three aspects of retouch variation: magnitude (marginal or invasive), extent (continuous or denticulated), and location (direct, inverse, alternating, and bifacial). According to Bordes (Bordes, 1951; Bordes & Sonneville‐Bordes, 1970), morphotypes were emic; i.e., configurations imbued with cultural significance. In contrast, (Debénath & Dibble, 1994; Dibble, 1995) interpreted retouched artefacts (mostly scrapers), as defined by Bordes (Bordes, 1961), as reflecting different stages along a continuum of morphological change resulting from the rejuvenation or reshaping of tool edges (see also the “finished artefact fallacy” (Davidson et al., 1989).

With the adoption of technological approaches based on the *chaîne opératoire* conceptual framework in the 1990 s, discussions about retouch variation and its meanings took new directions. The roots of Dibble’s perspective lie in the idea that variation in lithic assemblages resulted from adaptive strategies inherent to hunter-gatherers' modes of resource exploitation across the landscape (Binford, 1979a; Jelinek, 1976). In this view, lithic technologies were part of a dynamic technological decision-making process, involving the selection and integration of strategies for making, using, maintaining, transporting, and discarding tools, as well as the materials needed for their manufacture and maintenance (Nelson, 1991, p. 57). This aligns, to some extent, with the *chaîne opératoire* approach, which reconstructs sequences of actions, including knapping procedures, from raw material acquisition to tool discard. Binford had already pointed this out when he explained that such technological strategies are shaped by provisioning conditions (e.g., artefact production for future use, transport, maintenance, and recycling), all of which are closely tied to hunter-gatherer mobility (Binford, 1973, 1979a). He coined the term ‘curation’ to describe tools that are effective across multiple tasks (Binford, 1973, 1979a; Binford & Binford, 1966, p. 66). This represents a shift in archaeological reasoning from descriptive typology to processual analysis. At this point, a triad emerges: retouched tools, technological strategies, and the resulting artefacts (both products and byproducts).

**Fig. 1:**
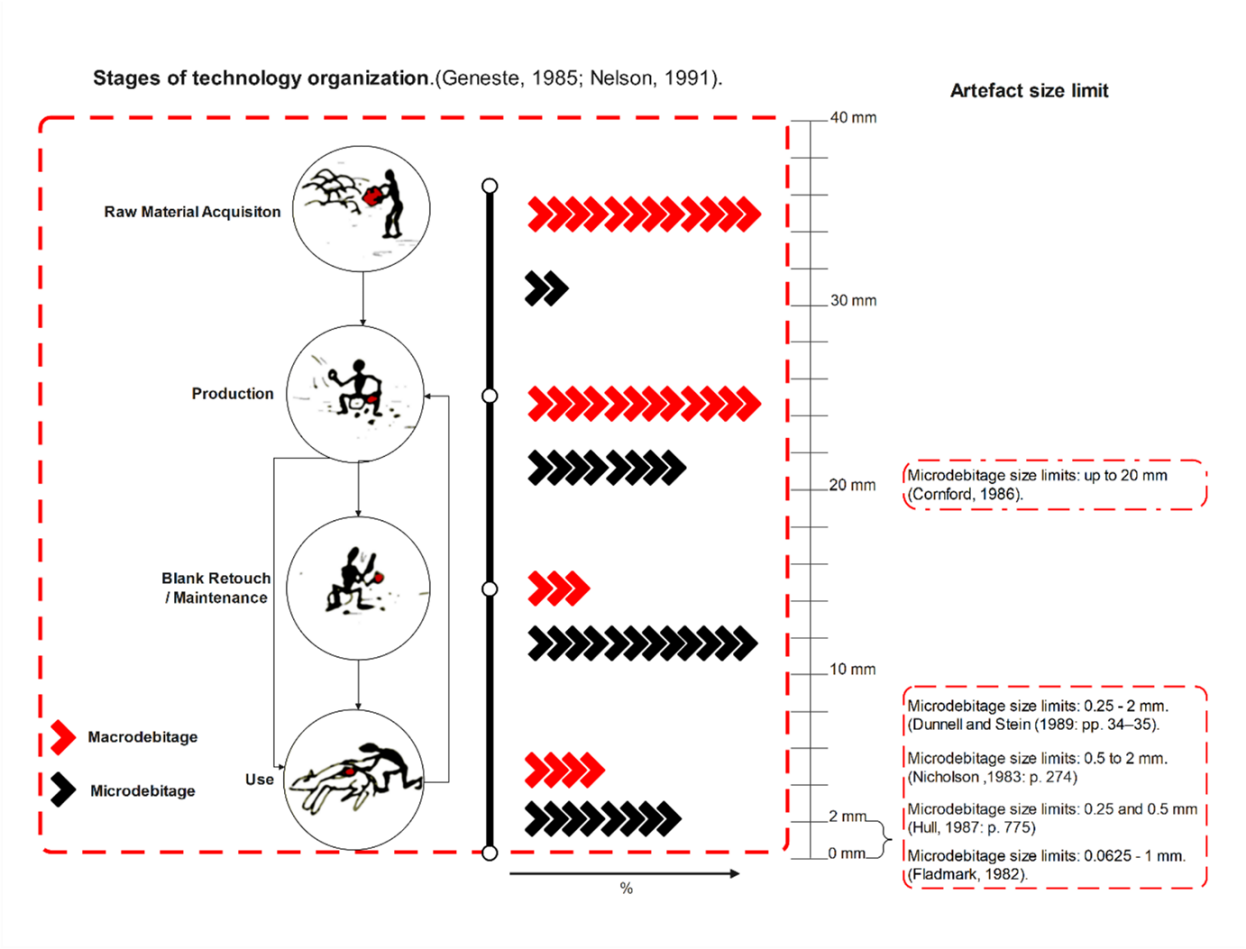
Macrodebitage and Microdebitage within the stages of technological organisation, with the highlight metrics and definitions of microdebitage

One of the key concepts linking these domains to curation is maintenance. Maintenance is intrinsically tied to curation, as the latter refers to strategies of caring for tools and toolkits to maximise their utility over time (Andrefsky, 2006, 2009; Cornford, 1986; Frison, 1968; Shott, 1986, 1989, 1994, 1996). According to Binford, curated technologies involve tools that are maintained through repeated use (Binford, 1973). Maintenance increases efficiency by extending a tool’s use-life relative to the energy invested in its manufacture. Frison (1968) highlighted the significance of use-life and resharpening, suggesting that maintenance behaviours reflect an investment in prolonging tool functionality, a concept aligned with curation. Bamforth (1986) explicitly argued that maintenance is a component of curation, proposing it as a response to shortages of raw materials. When raw materials are scarce, replacing worn tools is costly, making maintenance a more efficient strategy. Conversely, the frequency of tool maintenance is likely to decrease when raw materials are readily available. Shott (1996) redefined curation as the degree of use or utility extracted from a tool throughout its life. From this perspective, maintenance contributes directly to curation by increasing the total utility obtained before discard. Repair and reshaping enable a tool to be used for a more extended period and for more tasks, thereby fulfilling a greater portion of its initial utility. Maintenance includes human activities such as resharpening, repairing, and recycling to extend the usefulness of these items (Schiffer, 1976), which are achieved through edge modification techniques of retouching, reshaping, and rejuvenation. These activities often result in both intentional and unintentional production of byproducts, usually microartefacts (see Fig. 1).

Maintenance techniques have shaped the morphology of retouched pieces, which have transitioned from rigid typological constructs to dynamic, fluid products of economic decision-making (Geneste, 1985). The size of artefacts resulting from these maintenance actions poses a challenge. As these byproducts frequently fall into the category of microdebitage, they are often understudied or selectively sampled, focusing only on morphotypes deemed representative of specific technological signatures (Bourguignon, 1996a, 1996b; Cornford, 1986; Frick et al., 2024; Prévost et al., 2022). In this paper, we examine the technological interpretation and reconstruction of the operational sequence of maintenance techniques (retouching, reshaping, and rejuvenation) through two case studies from the Late Middle Palaeolithic of Armenia: Kalavan 2 and Ararat-1 Cave. Specifically, we propose and apply a new categorisation of microdebitage artefacts, classifying them into five modules (M0 through M4). These byproducts are viewed as dynamic expressions of decision-making, complementing static tool typologies, and offer a proxy for understanding the technological organisation of Pleistocene hunter-gatherers.

### Microdebitage Between Terminology and Technology

The technological method of analysis, rooted in the *chaîne opératoire* approach (Tixier, 1974; Tixier et al., 1980) views lithic production as guided by a cognitive plan translated into a conceptual framework and then operationalised into physical actions (Inizan et al., 1995:15). These actions leave physical traces on detached and worked pieces (e.g., dorsal scars, points of impact), which serve as markers to distinguish different technological stages. These stigmata are evident in both macro and microdebitage. As Soressi and Geneste (2011:337) emphasise, “*one advantage of the chaîne opératoire is its ability to define the 'temporality' and 'geography' of artefacts within the spatial and temporal context of knapping activities. Each object can be assessed in its processual context through experimentally verified attributes that clarify how prehistoric people fractured stone volumes to produce useable cutting edges, a kind of volumetric or 3D puzzle”.*

The recognition and identification of microdebitage are part of the broader classification of lithic artefacts. Artefacts of this particular category typically enter the archaeological record as small, shattered objects or microflakes produced during operations preceding the reduction sequence (Fig. 1). These fragments relate to the process of flake formation governed by fracture mechanics, with differing morphologies resulting from specific directions of flake initiation and spalling (Cotterell & Kamminga, 1987; Li et al., 2023a, 2023b; Speth, 1972, 1975). From this analytical standpoint, we refer to the intentional production of microdebitage as a result of maintenance activities. Microdebitage appears throughout most stages of the reduction sequence (Fig. 1), whether intentionally produced or not (Debénath & Dibble, 1994; Bertran et al., 2012; Bradbury & Franklin, 2000; Lin et al., 2016; Peña et al., 2022). At various stages of the reduction sequence, one can expect differing proportions of microdebitage, either as small, unintended byproducts of fracturing during technological gestures or as intentional products created for shaping, retouching, or rejuvenating a blank or tool. For instance, higher frequencies of intentionally produced microdebitage are expected during use, retouching, and maintenance stages rather than during initial blank production (Fig. 1).

Lithic classification systems are typically based on metric criteria that distinguish macrodebitage from microdebitage byproducts (Bar-Yosef, 1981; Odell, 2000, 2001, 2004, 1996), except in the case of bladelet production. To explore this further, we must first determine what threshold defines microdebitage. The size threshold has been inconsistently defined in the literature, with different terms and varying results, including microartefacts, microdebris, or chips (Clark, 1986; de la Torre et al., 2018; Dunnell & Stein, 1989; Fladmark, 1982; Frahm, 2016). These variations reflect size boundaries that range from 0.125 to 0.250 mm, up to 20 mm or more, depending on the assemblage context (e.g., chronology, site function), research feasibility, or analyst preference (see Frick et al., 2024).

Fladmark (1982:205) defined microdebitage as particles smaller than 1.0 mm resulting from deliberate lithic reduction. He noted that 1.0 mm represents the smallest particle size visible to the naked eye as a conchoidal flake and used it as a practical boundary between macrodebitage (1.0-3.0 mm) and microdebitage (< 1.0 mm). Many researchers subsequently revised their definitions and size thresholds accordingly. A recurring challenge is that very small flakes may resemble naturally occurring grains, making them difficult to distinguish from anthropogenic specimens (Clark, 1986; Schick, 1986; Vance, 1986).

Dunnell and Stein (1989) defined microdebitage as small, shattered objects or microflakes produced during percussion or pressure flaking. Cornford (1986), meanwhile, emphasised the relationship between artefact size and the ratio of waste (byproducts) to tools (products). He advocated the analysis of artefacts larger than 20 mm, whereas others argue for studying technological products irrespective of size.

The arbitrariness of size thresholds is illustrated in the work of Frison and Cornford. Both scholars established metric boundaries for technological analysis while examining links between microdebitage and stages in maintenance procedures. Frison (1968:149–152) described five types of retouch or sharpening flakes, derived from bifaces and scrapers, into which most unworked flakes could be categorised. Cornford identified longitudinal and transverse sharpening flakes, abbreviated as LSFs and TSFs, respectively. Although both researchers omitted prior micro and macrodebitage distinctions, they acknowledged the similar technological roles played by flakes across the reduction sequence (Cornford, 1986:341).

Other scholars distinguish between "use-flakes" detached during use and "retouch flakes" removed during formal modification (Hayes et al., 2014:78). (Chan et al., 2020) further divide flakes into resharpening or reworking flakes, depending on whether reshaping was intended. These classifications suggest diverse intentions, including use, retouch, reshaping, rejuvenation, or combinations thereof. Such gestures are part of the artefact's life history, contributing to maintenance and recycling (Bamforth, 1986).

Overlooking microdebitage due to its size omits a vital part of an assemblage behavioural signal. Conversely, a technological approach to microdebitage, rather than rigid size categorisation, enables the study of stigmata that inform the reconstruction of formation processes and link these to spatial and temporal aspects of dynamic knapping activities and technological decision-making.

This paper highlights edge modification techniques through retouch as proxies for maintenance activities, proposing a categorisation system that transcends size-based limits. We adopt Inizan et al.’s (1995) definition: “The term 'retouch' describes removals obtained by percussion or pressure, with the intention of making, finishing, or sharpening tools.” Consequently, the term "resharpening" is often used as a form of retouch (Iovita, 2011; Morales & Vergès, 2014) and is treated as such herein. In this way, the edge of a tool can be modified by retouching, reshaping, or rejuvenating. The term (re)touch implies the recurrence of the same touch on the edge. Gestures linked to (re)shaping can restore the original form of the edge (isometric) or alter it (allometric). (Re)juvenation techniques will produce a new edge. All such gestures result in byproducts with distinct technological stigmata, enabling their identification.

It is expected that patterned maintenance indices can be derived from formal tools in the archaeological record, whether via allometric (shape-changing) or isometric (shape-preserving) reduction (Iovita, 2014). Accordingly, byproducts from these actions can complement formal tool indices (Shott, 1994). While most studies focus on final products (e.g., Dibble, 1995), fewer examine byproducts (e.g., Bourguignon, 1996b; Cornford, 1986). Given the predominance of byproducts in assemblages, focusing on them offers a statistically robust sample that more accurately reflects technological events. These principles can support a classification system that reconstructs tool life histories through by-product analysis, creating a heuristic, behaviourally informative methodological framework (Iovita, 2011).

### Study Protocol

Our study protocol introduces an inclusive classification system for byproducts, focusing on their technological characterisation within the context of edge modification sequences (maintenance activities). In a previous work, an attempt was made to classify byproducts based on their technological features. Cornford (1986) has already identified and described such byproducts. As an attempt to gather more information about those flakes, he showed these to Harper Kelley and François Bordes in Paris, who explained that what they saw for them was *“r*e*trimmings of damaged larger tools. From this, it is clear that they recognized the marginal retouch to be prior, not subsequent, to the flakes' detachment.”*(Cornford, 1986, p. 337)*.*

The categorisation that we propose follows the same principles as flake identification, although we deal within the 5 to ≥ 20 mm size range. By identifying technical stigmata present on the lithic artefacts, caused by previous operations (i.e., the presence and location of scars on the dorsal face and the point of impact), we can place the artefacts in various steps within the space and the time of the flintknapping activity (*sensu* Soressi & Geneste, 2011). We classified these items according to the full attribute list that we used for the analysis of macrodebitage. Still, items smaller than 5 mm were counted but not thoroughly studied (Armagan, 2003, p. 103; Dunnell & Stein, 1989; Fladmark, 1982), as we were logistically unable to conduct the low-magnification microscopy needed for the identification of the lithic attributes. The development of this analysis protocol was conducted simultaneously as part of the general lithic attribute analysis (see raw data files in https://github.com/Nora-Arch/Moduleflakescategories). Data was gathered through an open-access entry software, E5 (https://github.com/surf3s/E5), with a personalised configuration file. All data analysis and plotting were processed using R, an open-source software, and JMP statistical software (Goos & Meintrup, 2015). A research compendium using the rrtools package by (Marwick et al., 2017, 2018), including detailed info on used packages, software versions, and raw and processed data, is available here: https://github.com/Nora-Arch/Moduleflakescategories.

To analyse whether lithic artefact attributes significantly varied across module categories (M0–M4), we utilised non-parametric statistical tests due to violations of normality assumptions indicated by Shapiro–Wilk tests. Non-parametric tests are robust alternatives suitable for archaeological data characterised by skewed distributions and unequal variances (Carlson, 2017). The Kruskal–Wallis H test, a non-parametric equivalent to ANOVA, was initially employed to assess overall differences in dorsal scar count, approximate area (length × width), and mass across modules. This test ranks data rather than assuming a specific distribution, making it ideal for detecting differences among multiple independent groups without requiring normally distributed data. Subsequently, significant results from Kruskal–Wallis tests were further investigated using Mann–Whitney U pairwise comparisons with Bonferroni corrections. This approach mitigates the risk of Type I errors from multiple tests, ensuring statistical rigour by adjusting the p-value thresholds according to the number of comparisons (Carlson, 2017). Boxplots visually supplement statistical results, clearly representing central tendencies, medians, and variability across modules, offering intuitive interpretations of statistical outcomes (Carlson, 2017). The box plots of module flake dimensions (Technological Length, Width, Thickness) demonstrate distinct patterns among module flakes across Ararat-1 Cave, Kalavan 2 T1, and Kalavan 2 T2.

### Baseline of the Module Identification

**Fig. 2:**
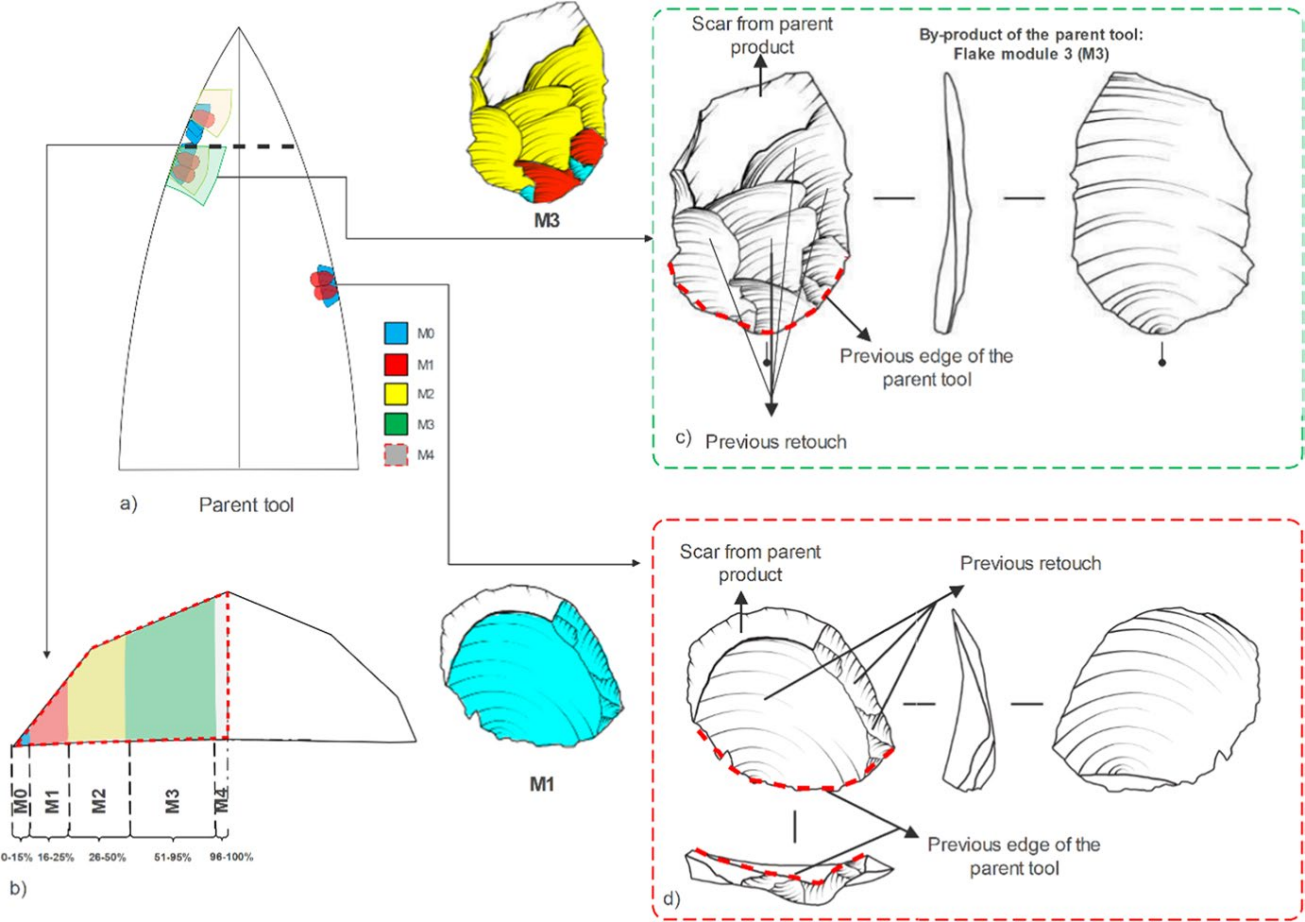
Schematic and archaeological representation of the criteria for the identification of a module flake. a. schematic representation of the parent tool. b. a cross-section of the “parent” tool with the schematic representation of the percentage ratio of increasing invasiveness. c. The main descriptive terms for module flake 3 are as follows: the colours indicate the different modules in b. d. Main descriptive terms for module flake 1. Note: Plain scar is the previous dorsal face of the initial edge (blank), so in the chronological sequence of flake removals, it is the first scar

Flake edge modification conforms to the same principle of flake formation and fracture mechanics (Cotterell & Kamminga, 1987). The proposed identification protocol aims for a detailed reconstruction of the modes of use of the piece, maintenance, and/or recycling (Bamforth, 1986). In a previous study (Malinsky-Buller et al., 2021), we identified these flakes as "shaping flakes," an inclusive term used to define all byproducts of tool modification. Their identification was based on the presence of retouch scars on their dorsal faces (that were generated by previous retouch or use), and the recognition of the point of intersection between the dorsal face and the platform, where it preserves the “parent” edge of the tool (Fig. 2). As the use/retouching/reshape/rejuvenation progresses, the technological signature of those removal leaves their mark on byproduct flakes (hereafter named “module flake”; see below). Most previously identified shaping flakes are < 20 mm in maximal dimension but are distinguished from “chips” that do not preserve visible retouch scars, or the recognition of a previous tool edge (e.g. (Malinsky-Buller et al., 2021)).

In our system, a module is a set of standardised parts or independent units (byproducts) that can be used to construct a more complex structure (i.e., retouched piece). The modules may consist of several distinct yet interrelated units (module flakes), which may be integrated into a holistic reconstruction of the tool's life history.

### Criteria

**Fig. 3:**
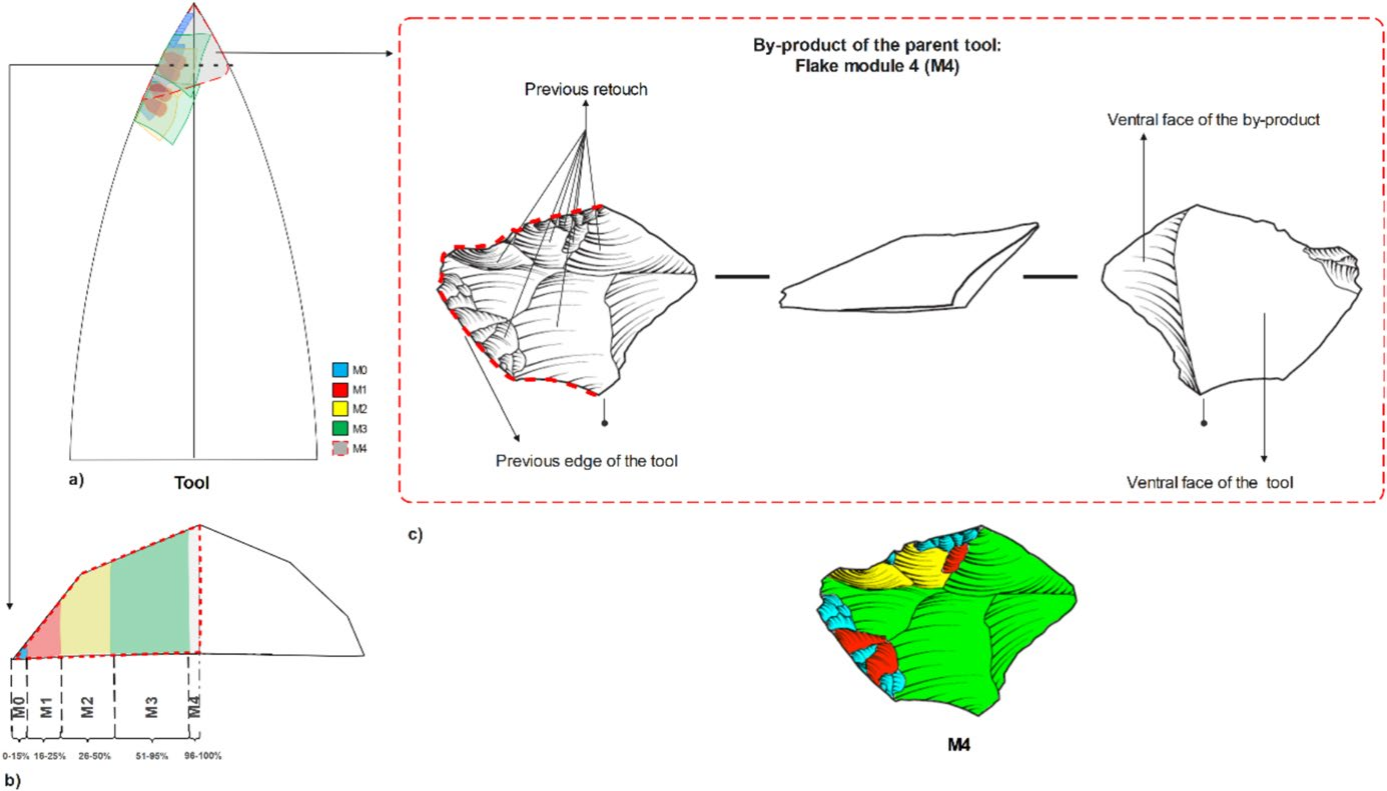
Schematic and archaeological representation of the criteria for the identification of a module flake. a. schematic representation of the parent tool. b. a cross-section of the retouched tool with the schematic representation of the percentage ratio of invasiveness. c. Main descriptive terms for module flake 4

Module flakes are identified and classified according to three variables: regularity, distribution, and invasiveness of the dorsal scars (maintenance techniques) in relation to the parent blank. The identification of a module flake is based on the following observations:

- The point of intersection between the dorsal and the platform preserves the “parent” edge of the tool- the previous working edge (Fig. 2/3 red dotted line).
- Identification of pre-retouch scars on the dorsal face (Fig. 2/3). As the use/retouching/reshaping/rejuvenation progresses, the technological signature of those removals leaves its mark on byproduct flakes, i.e., module flakes.
- Identification of the parent tool scar. When a flake is removed during retouching, it bears on its dorsal face the remnant of the scar(s) of the “parent” blank. This is a remnant of the “original” blank, and it can be plain or have one or more negatives of previous removals (rejuvenation stages on a tool).
- The pre-retouch scar direction in M0-M3 flakes is usually perpendicular to the striking platform; M4 may have several pre-retouched scar directions (see below).
- Invasiveness attribution. (Andrefsky, 2006; Clarkson, 2002; Eren & Sampson, 2009; Eren et al., 2005; Hiscock & Tabrett, 2010) calculate an invasiveness index for tools based on scars density per surface area upon the retouched tool. Here, we adapt this approach to an invasiveness percentage in relation to the surface area of the module flake (by-product of retouch). The invasiveness percentage is estimated based on the relationship between retouching scars and “parent scars”. The stratigraphy and superposition of those scars on the dorsal face of a module flake enable the distinction between the different invasiveness ratios, ranging from 1 to 100%, see Fig. 2/3b (See an example Fig. 3, c,d for the M3 and M1).

(see SOM).

According to these observations, we suggest categorising the byproducts of maintenance techniques into five module flakes of edge modification:

•M0: a flake with no previous edge modification. This flake does not show previous retouch scars. The main scar present is the remnant of the previous dorsal scar of the blank from the “parent” tool (similar to a Kombewa flake), directly from blank production, or the dorsal scar from the rejuvenation of the edge. It derives from the first edge modification, or the restart after edge rejuvenation. An estimated invasiveness ratio from 0–15% up to the blank ridge (Fig. 2/3).

**Fig. 4:**
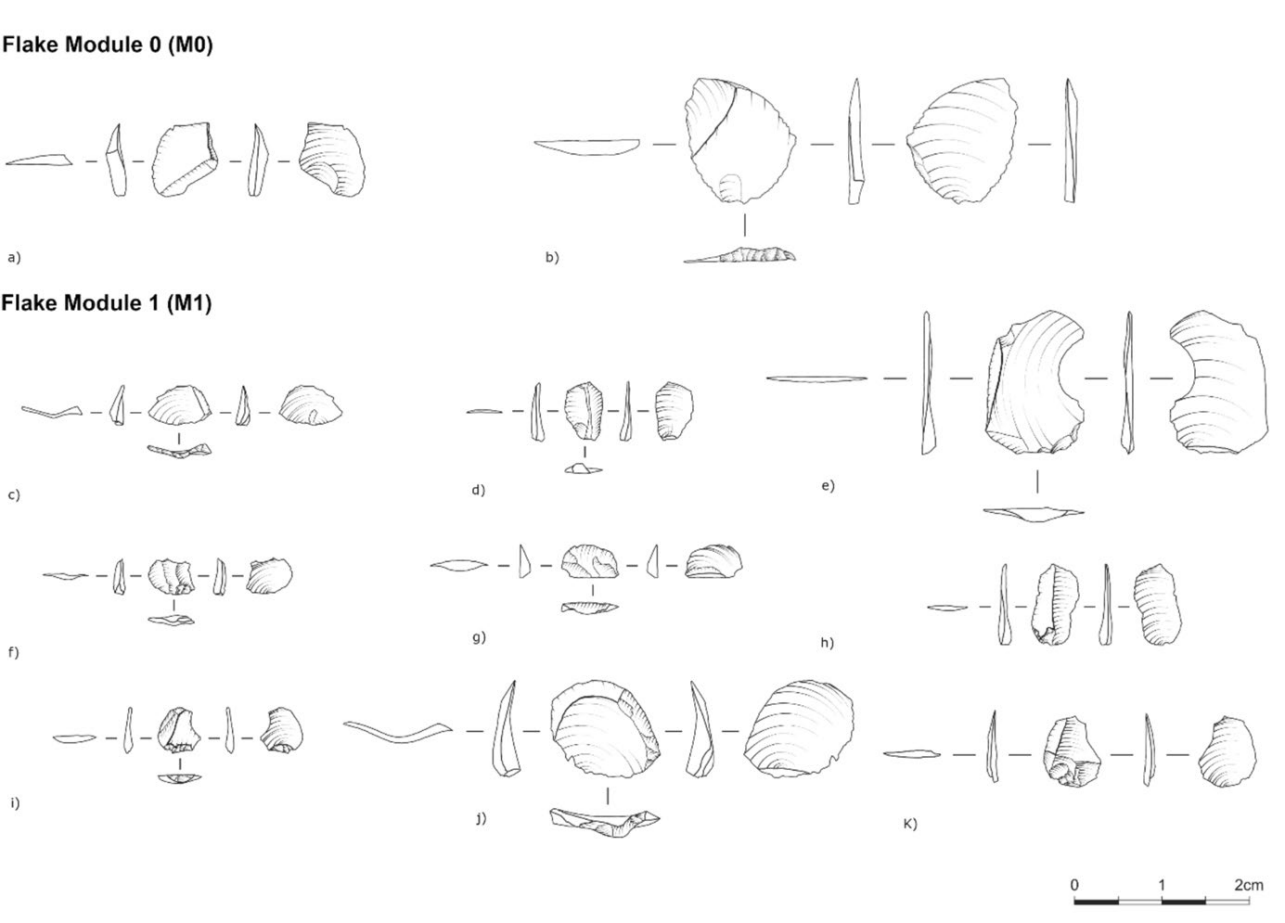
Archaeological examples of M0 and M1 flakes (from Ararat-1 Cave and Kalavan 2)

•M1: a flake stemming from the initial edge modification of M0. The initiation of retouch scars is associated with the striking platform of the module flake (i.e., the edge of the “parent” tool). Those scars stratigraphically overlay the “parent” tool plain scar(s), being a crucial criterion for their identification. An estimated invasiveness ratio from 16–25% up to the blank ridge. (Fig. 4).

**Fig. 5:**
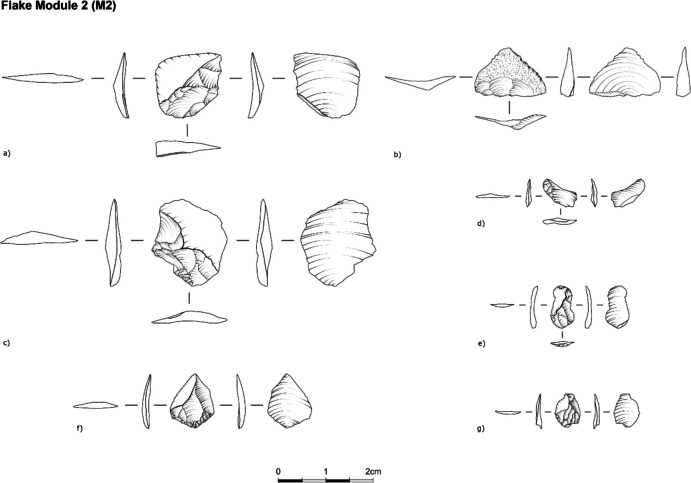
Archaeological examples of M2 flakes from Ararat-1 Cave and Kalavan 2(a, c-g—Obsidian; b -chert)

•M2: The scar pattern on the flake demonstrates an overlap of scars with remnants of M1, M0, and the plain scar(s) from the “parent” tool. An estimated invasiveness ratio from 26–50% up to the blank ridge (Fig. 5).

**Fig. 6:**
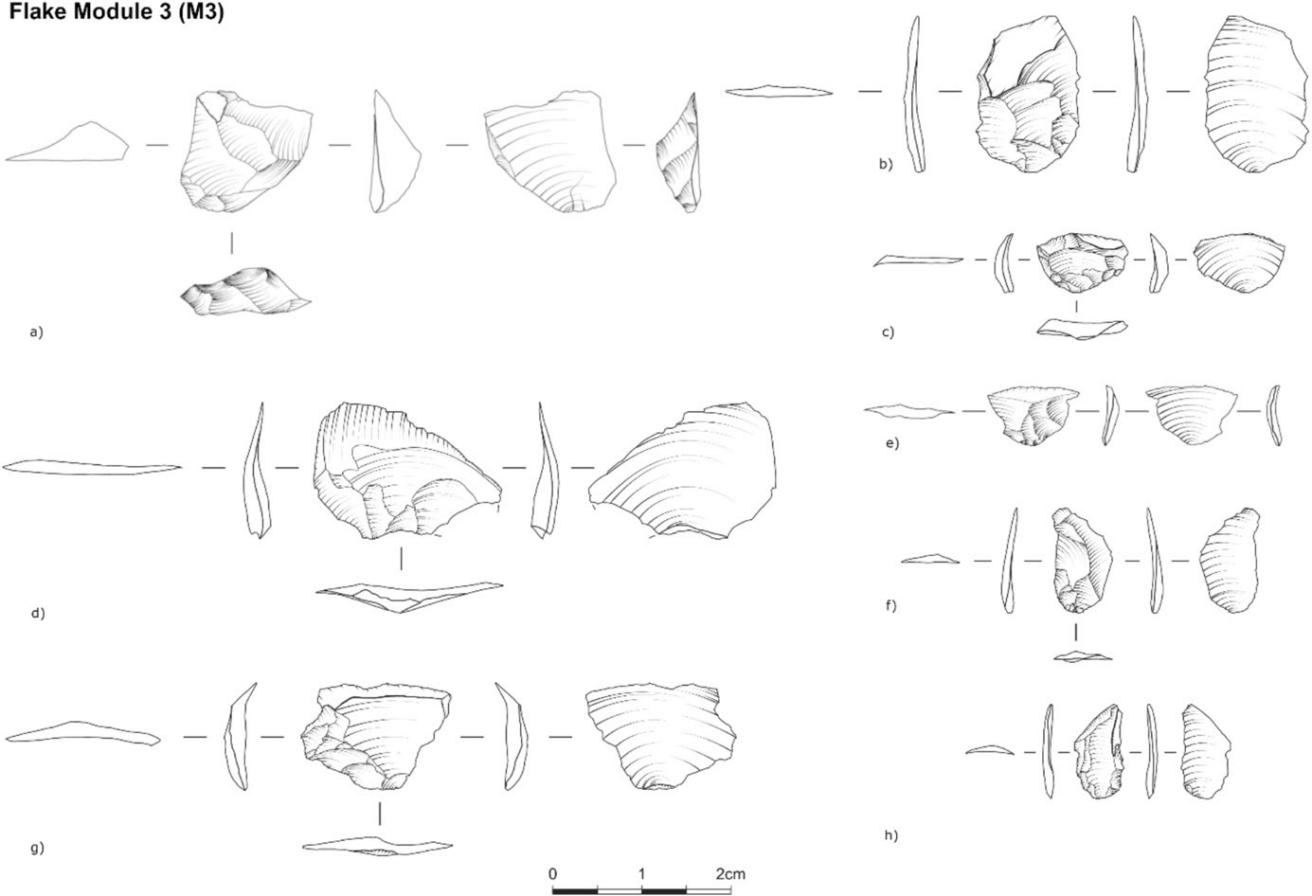
Archaeological examples M3 flakes from Ararat-1 Cave and Kalavan 2

•M3: The scar pattern on the flake shows an overlap of scars with remnants of the M2, M1, M0, and the plain scar(s) from the “parent” tool (Fig. 6). The estimated invasiveness ratio is from 51 to 95% up to the blank ridge.

**Fig. 7:**
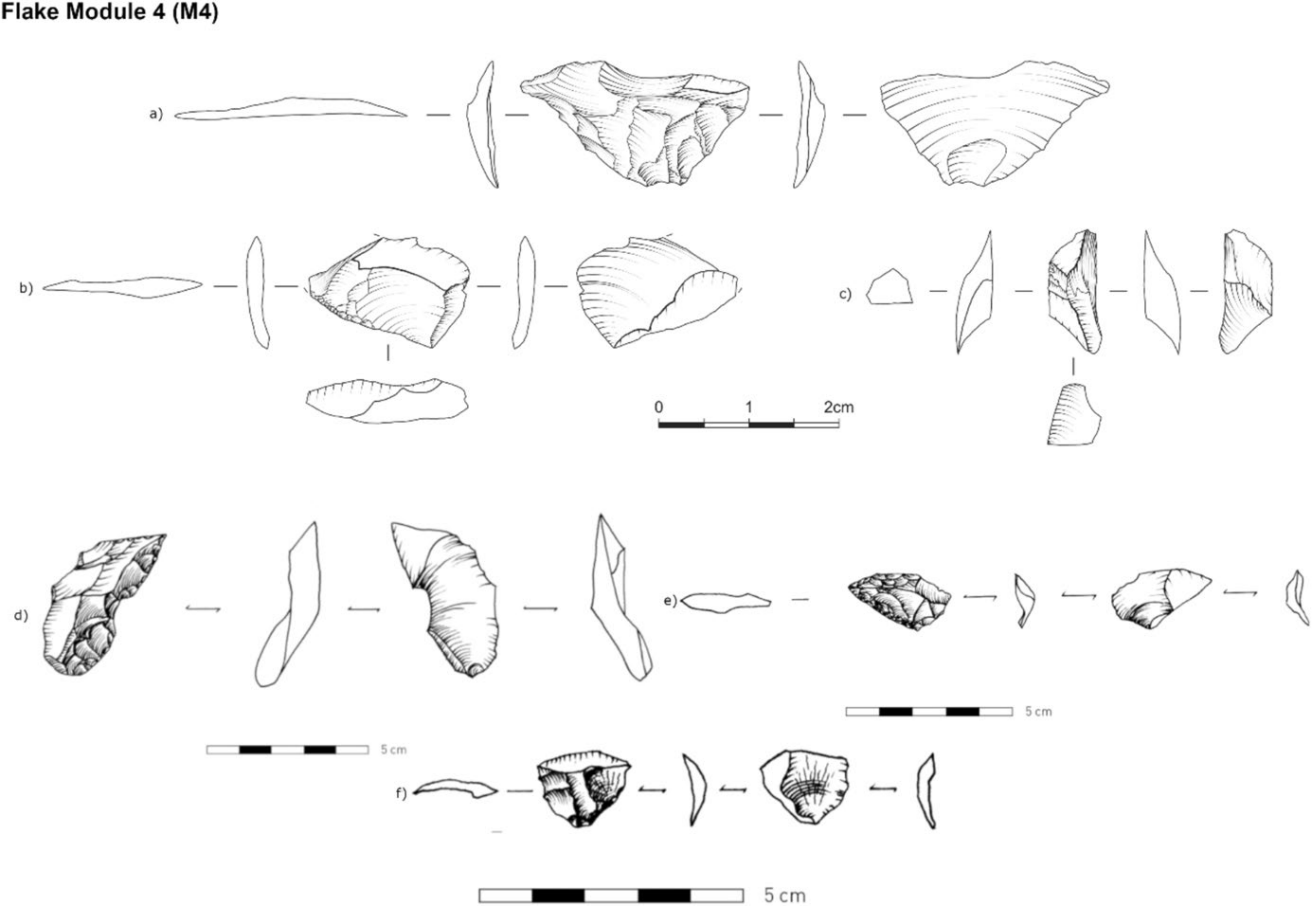
Archaeological examples of M4 flakes from Ararat-1 Cave and Kalavan 2 (module flakes d/e/f were drawn by Phill Glauberman)

•M4: The scar pattern on the flake shows an overlap of scars with remnants of the M3, M2, M1, and M0 and the plain scar(s) from the “parent” tool. The range of estimated invasiveness ratio values is from 95–100% of retouch invasiveness (Fig. 7). The presence of two ventral faces (the “new” and the “older” from the original tool removed – Fig. 3) can be used to distinguish M4 flakes from the rest.

By removing an M4 flake, the knapper creates a new clean edge by reshaping/rejuvenating rather than using a retouching technique (M0-M3). An M4 flake removes part of the tool's edge to either maintain the same shape or modify it (allometric vs. isometric morphologies (Iovita, 2014). The presence of two ventral faces can distinguish M4 flakes from other module types. Similar technological pieces were already identified throughout the Palaeolithic in different contexts and regions, either in bifacial or unifacial shaping (Bourguignon, 1996a, 1996b; Lamotte, 1999; Verjux & Rousseau, 1986). For example, Cornford (1986) pointed out that the removal of a Long Sharpening Flake (LSF), which we term an M4 module flake, creates a new edge with the greatest possible length and sharpness on the parent tool. The uniqueness of this type made it more distinguishable when analysing the lithic assemblages in comparison to M0-M3 (Conard & Adler, 1997; De Loecker, 2006; Fonton et al., 1991; Frick et al., 2017; Malinsky-Buller, 2014; Roebroeks et al., 1988, 1997); Zaidner & Grosman, 2015).

**Fig. 8:**
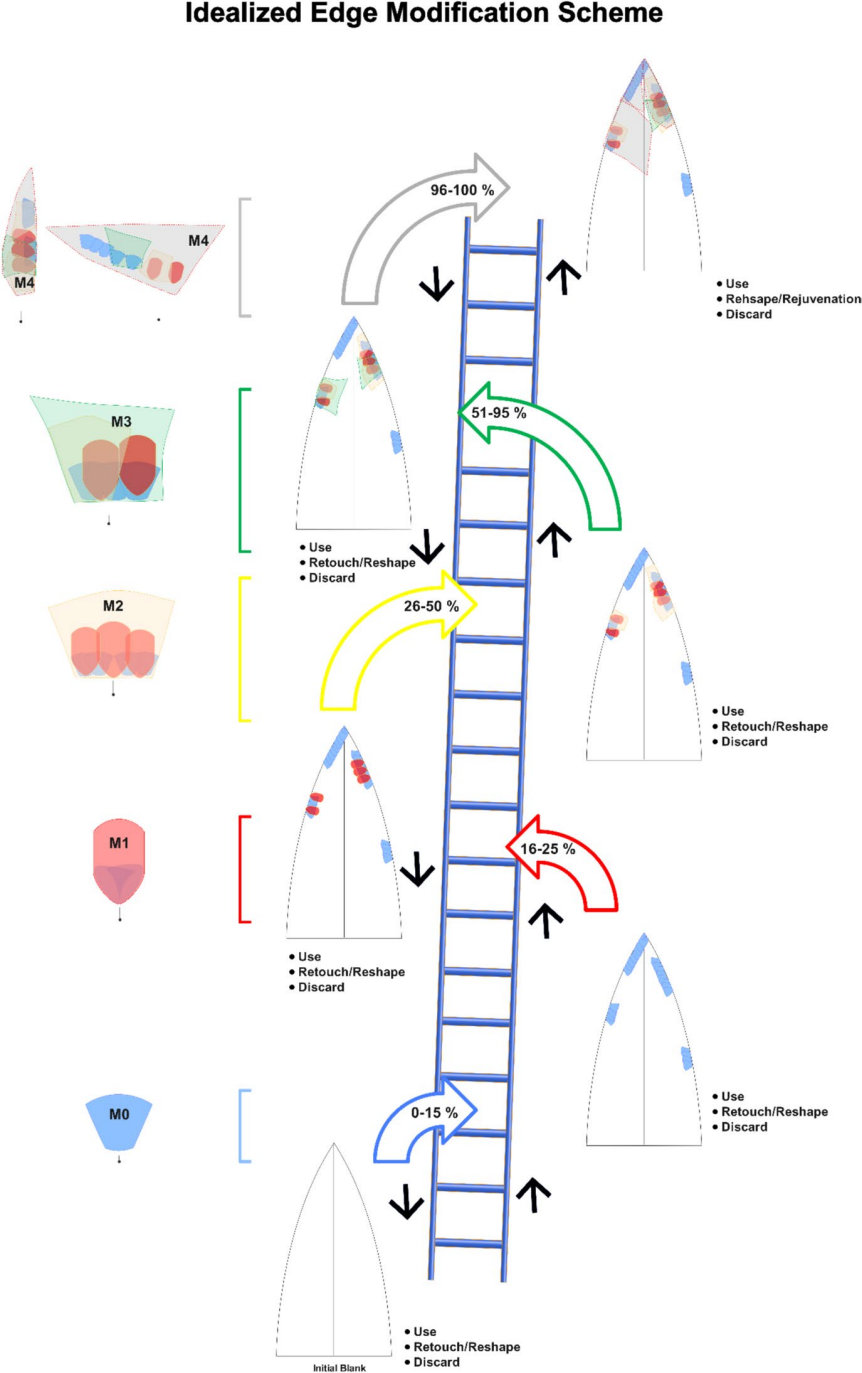
Idealised edge modification scheme illustrating the module progression of lithic tool use, retouch, and discard across edge modification modules (M0–M4). The vertical ladder represents increasing stages of utilisation of the edge (0–100%), with curved arrows denoting estimated use-life ranges for each module, and the black arrows representing transcend stages of a tool maintenance, for example, after the removal of several M1s, the knapper can apply an M4 (see Lateral Tranchet Blow (LTB) at Nesher Ramla (Prévost et al., 2022). Modules correspond to characteristic retouch/reshaping/rejuvenation patterns, with M0 reflecting minimal use and M4 representing heavily modified tools nearing the saturation point. The model emphasises cyclical behaviours of use, retouching/reshaping, and potential rejuvenation before final discard

Therefore, using the terminology of module flakes, we aim to deviate from a linear and staged classification sequence, where each step depends on the previous one. Rather, we want to create a dynamic classification (Fig. 8).

The module flake system is designed to capture progressive maintenance activity. We formulated explicit expectations about how the categories should behave and what they would mean.

- Dorsal scar accumulation, later modules (M3-M4) should show higher numbers of dorsal scars than earlier modules (M0-M1-M2). This reflects the cumulative nature of edge modification either by use or maintenance techniques, where repeated removals leave increasingly complex scar patterns. If this pattern is present, it indicates that module flakes successfully capture the additive process of edge modification of the tool.
- Material investment, later modules should show greater mass values, since progressively invasive retouch requires the removal of more material. Mass, therefore, serves as an independent proxy for the intensity of maintenance. If this pattern holds, it validates the use of module flakes as a quantitative measure of retouch investment, beyond morphology alone.
- The same is valid for the first approach by the knapper to a “clean” blank/edge when we have higher frequencies of M0 flakes. The higher signal of M0 flakes indicates the exploitation of an edge that was not modified (i.e., the first edge modification) or is in a second or more stages of rejuvenation. This pattern can be explored by the presence of the parent scar of the blank at the module flakes, in association with the dorsal scar sections. Both higher signals of M0 and M4 flakes help us to identify when tools with no retouch are being used and maintained. All these assumptions have more in-depth resolution when we can differentiate the Raw Material Units (RMU) and start building a use-life story of each. In so doing, when we integrate module flake categories into the lithic attribute analysis based on the technological approach, disregarding dimensions, we can explore questions related to tool maintenance activities. This, in return, provides us with a proxy to quantify the resolution of past hunter-gatherers' mobility and their degree of tool use-life.

### Case Studies

The two case studies presented, Ararat-1 Cave and Kalavan 2 -T1/T2, show high frequencies of artefacts that would typically fall within the category of chips/debris in a size-oriented analysis. These characteristics are known from other Middle Palaeolithic archaeological sites in the southern Caucasus (e.g., Gasparyan et al., 2014; Glauberman et al., 2020; Malinsky-Buller et al., 2021; Yeritsyan, 1972). By utilising the novel categorisation of the module flake in both Kalavan 2 and Ararat-1 cave assemblages, we demonstrate the utility of the high-resolution analysis of microdebitage assemblages in terms of provisioning behavioural signatures.

### Kalavan-2

The open-air site of Kalavan 2 (UTM 40 T 3821 m E, 450551 m N, 1640 m asl) is located on the northern slopes of the Areguni Mountains at an elevation of. ca. 8 km north of the shores of Lake Sevan. The chronology of the site is based on fifteen post-infrared infrared stimulated luminescence (pIRIR) samples (Malinsky-Buller et al., 2021) as well as C14 ages from micromammal remains (Rogall et al., submitted). The age range of the main archaeological layers is between 60- 45ka BP (early MIS 3). In total, 2075 lithic artefacts (of which 49 are retouched tools and 819 module flakes) from 13 sedimentary units were retrieved. Three main archaeological layers were exposed, Unit 1b in T1 and Units 4 and 7 in T2, with 990 lithic artefacts (of which 38 are retouched tools and 275 module flakes). The recovered lithic artefacts were made from seven raw materials: obsidian, basalt, dacite, welded tuff, chert, limestone, and an unidentified metamorphic rock. The frequencies of raw material types vary from unit to unit. Obsidian is the most common material. There are no indications for the first stages of core preparation and reduction, while the debitage consists mainly of flakes < 2 cm. Retouched obsidian pieces present an extremely narrow range of tool types, the majority falling within retouched points or convergent scrapers. The non-obsidian component exhibits more substantial indications of core reduction, including flakes, Kombewa flakes, and Levallois flakes (Ghukasyan et al., 2010; Malinsky-Buller et al., 2021).

### Ararat-1 Cave

Ararat-1 cave (39.851 N, 44.769 E, 1034 m asl) is situated 2 km east of the town of Ararat on the northeast margins of the Ararat Depression. The two archaeological horizons are radiometrically dated by 12 post-infrared infrared stimulated luminescence (pIRIR) samples and a single C14 sample to a range between 50- 35ka BP (Oikonomou et al., 2025; Sherriff et al., 2024). The lithic assemblage of Ararat-1 is mainly comprised of chert and obsidian, with mafic lava, chalcedony, and quartzite represented by a few isolated pieces. The total assemblage amounts to 1770 artefacts, of which 37 are retouched tools and 680 (38% of the assemblage) are module flakes smaller than 10 mm (108 obsidian and 26 in chert) (Nora et al., 2025a; Frahm et al., 2025). There are a few indications of obsidian blank production at the site (very few cores or primary elements); in this raw material, it is mainly the last stage of the reduction sequence that is represented in the assemblage: use, maintenance, and rejuvenation (as defined/described by Geneste, 1985; Nelson, 1991). Typologically, the retouched pieces are dominated by several types of scrapers and retouched flakes. The techno-typological composition of the chert component attests to initial stages of the reduction sequence, including cores and cortical elements (Nora et al., 2025a). Adding to the regular attribute analysis, we incorporated a proposed module for microflake categorisation (M0–M4), we analysed key lithic attributes such as technological length, technological width, thickness, number of dorsal scars, and raw material distribution across different modules to show the difference between them and what they represent behaviourally.

## Results: Lithic Attribute Analysis across Modules

### Technological Length/Width/Thickness:

#### Kalavan 2 T1

**Fig. 9:**
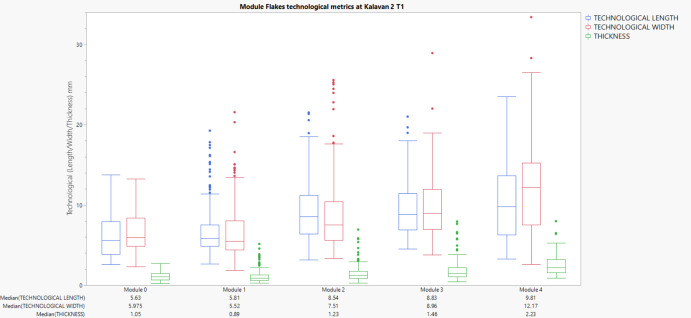
Box plots displaying the metric attributes (Length, Width, Thickness) of lithic flakes across edge modification modules at Kalavan 2 T1. Each chart represents a distinct module (M0-M4), with individual observations for the respective medians

First modification module flakes (M0–M1) display consistently smaller sizes, with M0 median dimensions of 5.63 mm (length), 5.97 mm (width), and 1.05 mm (thickness). Module 1 medians are similar at 5.81 mm (length), 5.52 mm (width), and 0.89 mm (thickness). At Module 2, we see an increase in flake size (median length 8.54 mm, width 7.51 mm, thickness 1.23 mm), continuing progressively to Module 4, where module flakes reach median lengths of 9.81 mm, widths of 12.17 mm, and thicknesses of 2.23 mm. (Figure 9).

#### Kalavan 2 T2

**Fig. 10:**
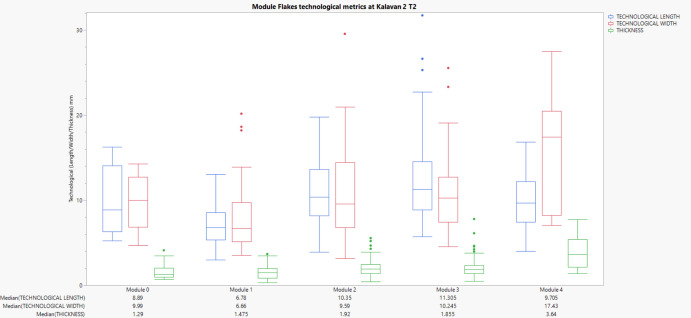
Box plots displaying the metric attributes (Length, Width, Thickness) of lithic flakes across edge modification modules at Kalavan 2 T2. Each chart represents a distinct module (M0–M4), with individual observations for the respective medians

Flakes from Kalavan 2 T2 show relatively larger dimensions compared to Kalavan 2 T1 across most modules. Median values for Module 0 are 8.89 mm (length), 9.99 mm (width), and 1.06 mm (thickness). Module 1 exhibits a size reduction (length: 6.78 mm, width: 6.66 mm, thickness: 1.31 mm), followed by clear increases in subsequent modules. Module 4 flakes have larger median dimensions of 9.71 mm (length), 17.43 mm (width), and 2.87 mm (thickness). (Figure 10).

#### Ararat 1 Cave

**Fig. 11:**
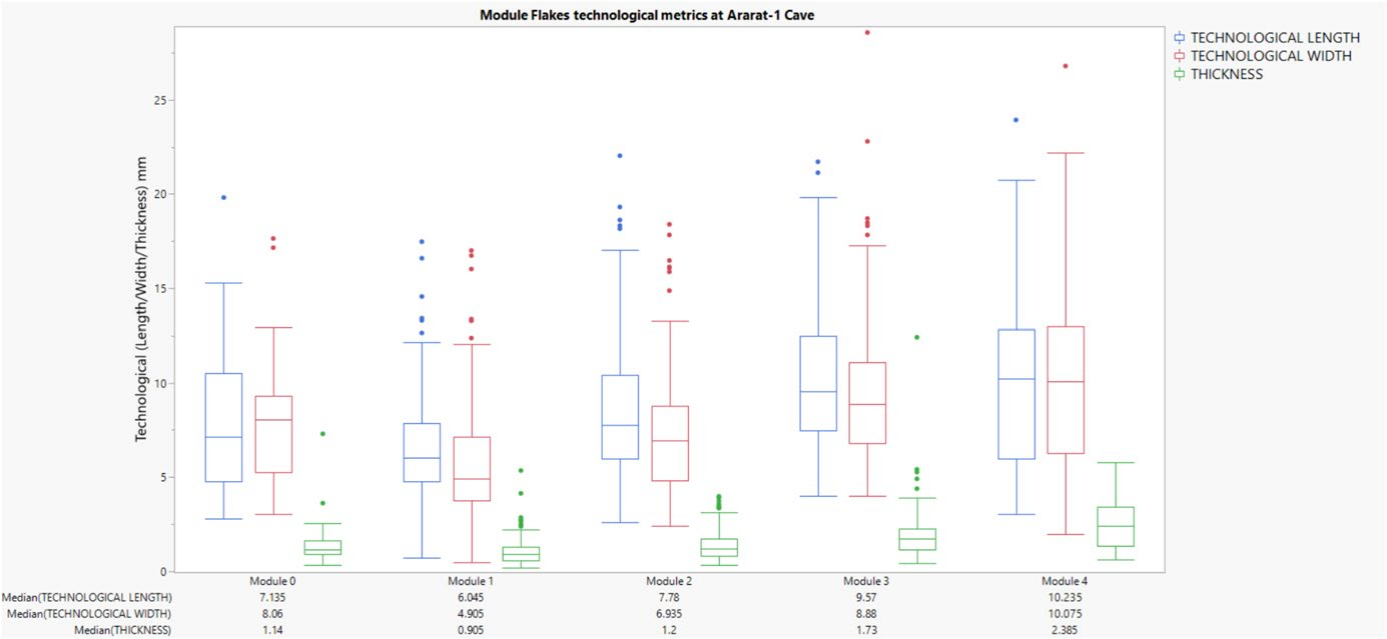
Box plots displaying the metric attributes (Length, Width, Thickness) of lithic flakes across edge modification modules at Ararat-1 Cave. Each chart represents a distinct module (M0–M4), with individual observations for the respective medians

Flakes from earlier modules (M0–M2) exhibit smaller and thinner dimensions. For instance, M0 flakes have median values of 7.14 mm (length), 8.06 mm (width), and 1.14 mm (thickness). Module 1 flakes show smaller removals with median dimensions of 6.05 mm (length), 4.90 mm (width), and 0.90 mm (thickness). However, dimensions notably increase in later modules, with M4 flakes reaching median lengths of 10.24 mm, widths of 10.08 mm, and thicknesses of 2.39 mm. (Figure 11).

### Statistical Results: Number of Dorsal Scars, Area, and Mass

**Fig. 12:**
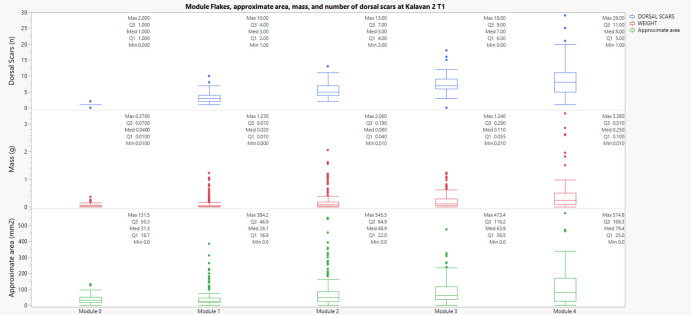
Relationship between the number of dorsal scars and approximate area (mm^2^) and mass (g) of module flakes across edge modification modules at Kalavan 2 T1. Each panel corresponds to a different variable through the module's flakes categories

In Kalavan 2 T1, all three Kruskal–Wallis tests were highly significant: approximate area (χ^2^ = 154.61, p < 0.001), number of scars (χ^2^ = 409.92, p < 0.001), and mass (χ^2^ = 154.96, p < 0.001). Pairwise Mann–Whitney U tests showed that flakes from Module 0 were significantly different in area and mass from those in Module 4. In contrast, Module 1 differed strongly from Modules 3 and 4 across all three variables. Additionally, Module 2 stood out from Modules 0 and 1, demonstrating that even at intermediate stages, significant increases in dorsal scar frequency, surface area, and weight are already evident. These findings reflect a progressive, stepwise intensification of flake modification. (Figure 12).

**Fig. 13:**
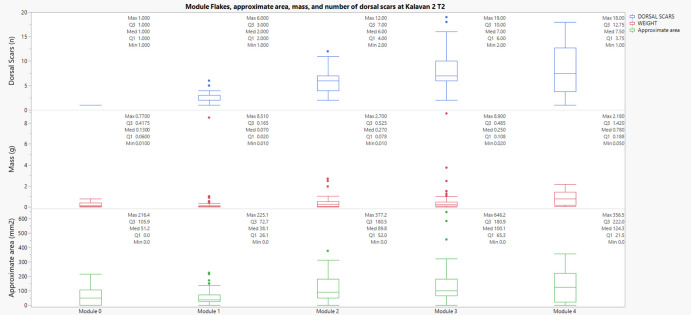
Relationship between the number of dorsal scars and approximate area (mm^2^) of module flakes across edge modification modules at Kalavan 2 T2. Each panel corresponds to a different variable through the module's flakes categories

In Kalavan 2 T2, the Kruskal–Wallis tests also revealed highly significant results for area (χ^2^ = 58.06, p < 0.001), scar count (χ^2^ = 137.86, p < 0.001), and mass (χ^2^ = 56.31, p < 0.001). Pairwise comparisons showed that the area significantly increased from Module 1 to Modules 2, 3, and 4. Scar count increased significantly between Module 0 and Modules 1 and 4, reinforcing the stratigraphic accumulation of retouch scars through successive stages. Mass differences were less frequent but became notable in the transition to Module 4. These results point to both additive scar layering and material investment through advanced retouching. (Figure 13).

**Fig. 14:**
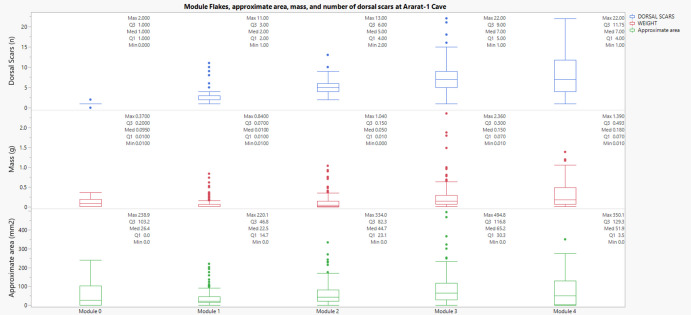
Relationship between the number of dorsal scars and approximate area (mm^2^) of module flakes across edge modification modules at Ararat-1 Cave. Each panel corresponds to a different variable through the module's flakes categories

In Ararat 1 Cave, the Kruskal–Wallis tests yielded significant results for all three variables: approximate area (χ^2^ = 129.50, p < 0.001), number of dorsal scars (χ^2^ = 299.54, p < 0.001), and mass (χ^2^ = 115.57, p < 0.001). Pairwise Mann–Whitney U comparisons for area revealed highly significant differences between Module 1 and Module 2, Module 3 and Modules 1 and 2, and Module 4 and Modules 1 and 2. Scar count comparisons indicated that Module 4 had significantly more dorsal scars than both Module 0 and Module 2. For mass, significant differences were detected between Module 4 and earlier modules, especially Module 0. These results could indicate that retouch intensity, as measured by increased scar count and mass, correlates with later module stages, confirming morphological and volumetric divergence from early-stage module flakes. (Figure 14).

Across all three sites, Kruskal–Wallis and Mann–Whitney U tests confirm that flake morphology, scar density, and mass vary significantly across module categories. Most of the differentiation is visible between early (M0–M1) and advanced (M3–M4) modules, with intermediate stages showing incremental but statistically significant changes. These results demonstrate that the module classification effectively describes technological and behavioural distinctions, aligning closely with principles advocated by Carlson (2017) regarding quantitative archaeological data analysis. Module categories reflect cumulative technological processes and retouch investment on tools, rather than arbitrary distinctions.

### Lithic Raw Materials

The integration of the classification of module flakes and their frequencies with raw material units enables us to decipher the fragmentation of the reduction sequence, specifically when looking at the phases of tool maintenance and use (Turq et al., 2013).

**Fig. 15:**
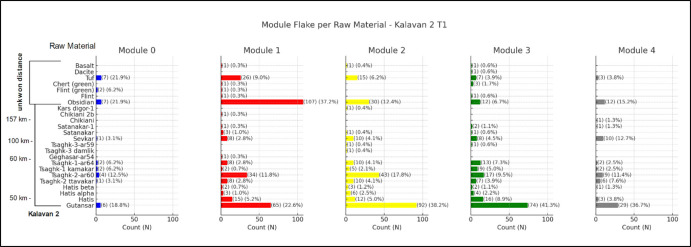
Distribution of module flakes by raw material at Kalavan 2 T1. Each bar represents the count and percentage of flakes made from a given raw material within each module (Modules 0–4). Raw materials are listed along the y-axis, ordered by approximate source distance from the site (left). The chart illustrates both the diversity of raw materials and their varying frequencies across modules

In the Kalavan 2 T1 assemblage (Fig. 15), the Gutansar (47 km) obsidian appears frequently in Modules 0 to 4. Hatis (50 km) has no representation in Module 0, while it is more prominent in Modules 1, 2, and 3, with a few M4 pieces. The outliers in this plot are the Chikiani source (140 km), represented by a single M4 flake, Chikiani 2b on M1, and Kars Digor (158 km), which is represented by a single M2 occurrence. Tsaghkunyats sources (60 km) are present in all modules. The same tendency is visible for non-obsidian raw material tuff. Module flakes made on flint and chert (green) are relatively rare, appearing in small amounts in Modules 1 and 2.

**Fig. 16:**
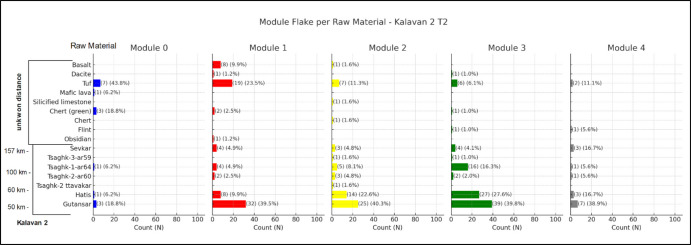
Distribution of module flakes by raw material at Kalavan 2 T2. Each bar represents the count and percentage of flakes made from a given raw material within each module (Modules 0–4). Raw materials are listed along the y-axis, ordered by approximate source distance from the site (left). The chart illustrates both the diversity of raw materials and their varying frequencies across modules

At Kalavan 2 T2 (Fig. 16), obsidian sources from Gutansar (47 km) and Hatis (50 km) are a significant raw material, and their presence is particularly prominent in Module 3. Tsaghkunyats sources (60 km) have almost no representation on M0. In modules 1 to 3, the frequencies are up to 2%, followed by a single example in Module 4. Among the non-obsidian flake modules, tuff stands out. It is particularly dominant in M0, accounting for 43.8% of the material in this module, and continues to be represented across all modules, including M4. This consistent presence of tuff from M0 to M4 suggests its sustained utility throughout various stages of retouching and modification. Basalt and dacite are only minimally represented, primarily in the earlier modules. As for flint, this raw material has no representation in the first three modules, but it appears as isolated instances in the M3 and M4.

**Fig. 17:**
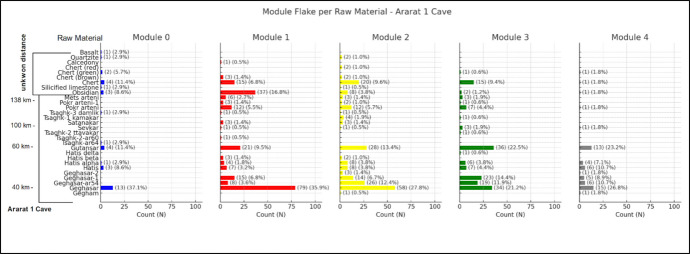
Distribution of module flakes by raw material at Ararat-1 Cave. Each bar represents the count and percentage of flakes made from a given raw material within each module (Modules 0–4). Raw materials are listed along the y-axis, ordered by approximate source distance from the site (left). The chart illustrates both the diversity of raw materials and their varying frequencies across modules

Ararat-1 Cave (Fig. 17) exhibits some unique tendency. Obsidian source Geghasar (35–40 km), a prominent raw material at this site, is highly concentrated in Modules 1 and 2. Gutansar (60 km) also shows a similar pattern, but its peak occurs in Module 3. Hatis (50 km) and its variants (Hatis alpha, beta, and gamma) appear consistently across all module categories but are less frequent in the earlier stage (Module 0). Items from the Arteni sources (100–130 km) appear in Modules 1 to 3, with sporadic appearances in Modules 0 and 4. Non-obsidian raw materials, such as chert, basalt, and mafic lava, are rare in this assemblage and only show marginal appearances in Modules 0, 1, and 2.

## Discussion: Behaviour beyond module flakes

The study of retouched pieces plays a key role in the systematics of the Palaeolithic record. As best exemplified by the nomenclature developed by Bordes (Bordes, 1953, 1951, 1961), pre-determined morphotypes shaped by retouching arguably conveyed cultural significance. In parallel, the perspective originally put forward by Frison (1968), later Jelinek (1976), and culminated in the works of Dibble and his students (Dibble, 1985, 1995), suggested that those same types were stages along a continuum of size change, that crossed retouching, reshaping, and rejuvenation stages. At the same time, Lewis Binford (Binford, 1973, 1979) positioned the theoretical foundation of curation by stating that those maintenance techniques were part of a bigger concept, which emphasises maximisation of raw material utility through maintenance and recycling linked with procurement strategies. Yet despite the theoretical richness of these perspectives, their empirical application remains debated, beginning with the most fundamental issue, the basic unit of analysis. For Shott (1989, 1996) and Dibble (1995), this unit was the retouched tool. However, Shott (1989:24, 1996) adds a twist on the concept definition, *“The degree of curation, defined as the degree of use or utility extracted, expressed as a relationship between the tool's initial maximum utility and how much of that utility is realised before discard.”* In contrast, (Barton & Riel-Salvatore, 2014; Clark & Barton, 2017) proposed an assemblage-level approach (WABI) to explore curation, against the expedient technologies. Despite selecting different analytical units, those methodologies and empirical toolkits aim for similar “higher-level” questions, such as the reconstruction of the mobility patterns of the group and the extent of occupation within a locality, as well as resource exploitation intensity. Usually, analysts choose to focus exclusively on studies that link identifiable artefacts, commonly macrodebitage artefacts, in most cases attributed to tools with non-local raw materials that could be brought from localities that exceed the daily exploitation territory (DET) to propose mobility models (Bailey & Davidson, 1983; Kuhn, 1992, 1995; Vita-Finzi et al., 1970). Nevertheless, other studies (Ekshtain et al., 2017; Féblot‐Augustins, 2009; Hovers, 2009; Mitki et al., 2021) challenge these assumptions by reevaluating the analytical unit through the examination of different proxies, such as raw materials composition, artefact size, different frequencies of typological items, formal vs. non-formal cores, and the relation between lithic and faunal remains, in order to propose mobility or procurement strategies.

Building on this, our paper shifts the unit of analysis to the byproducts of maintenance techniques, disregarding their size, as a new proxy for identifying maintenance strategies of tools that have been used and reused or removed from the site (ghost tools). This new approach also provides a fresh perspective on the intensity of edge modification techniques such as retouching, reshaping, and rejuvenation. Our application of the concept of curation aligns with that of Shott, in that we regard it as a behaviour designed to extend an artefact's use-life. This perspective enables us to refine methods for documenting and quantifying the degrees of extension of a retouched tool's use-life. Our proposed nomenclature and categorisation protocol for "module flakes" are based on fundamental principles of fracture mechanics that apply to all artefacts, regardless of size. We emphasise identifying key diagnostic features in lithic studies, acknowledging that tools are manufactured through processes of retouching, reshaping, and rejuvenation. From a unified analytical foundation, we apply the same technological attribute analysis to microdebitage artefacts as we do to macrodebitage, serving the same analytical purposes.

We establish here a module categorisation of flakes to overcome the inconsistencies in the literature. “Module flakes” have been identified through several periods and contexts on lithic assemblages from the Palaeolithic to the New World in the Late Prehistoric context (Bourguignon, 1996a, 2001; Cornford, 1986a; Frison, 1968; Hays, 1992; Touzé, 2018). Synonym nomenclatures are available, either as the proposed five categories of retouch and sharpening flakes by Frison (1968), or the attribution of the LSF, TSF flakes of Conford (1986), or the several phases of decortication and post-decortication suggested by (Jérémie & Vacher, 1992). For the latest phase of rejuvenation of retouched tools (our “M4”), a wide range of synonyms were applied, all depending on the directionality of the removals, such as Tranchet Blow, or Lateral Tranchet Blow (LTB) at Nesher Ramla (Prévost et al., 2022), or the chanfren (Burney, 1967), for more morphological differences, see (Frick et al., 2024). We argue that most of these different flake typologies that are technological flakes related to edge modification are targeting specific human gestures of retouch/reshape and rejuvenation applied to the tool (Tixier et al., 1980). Thus, that can be perceived as a unit instead of several typologies. Despite the abundance of terminologies and contextual examples, a unified technological framework for understanding these flakes has yet to be fully articulated.

The progressive modification of a blank through retouching and reshaping is the main proxy used here to assess the use-life of a lithic tool. Archaeologists have made significant efforts to develop new quantitative methods based on stone tools to understand lithic reduction, more specifically, retouch intensity (Clarkson, 2002; Davis & Shea, 1998; Dibble, 1987, 1995; Eren & Prendergast, 2008; Eren & Sampson, 2009; Eren et al., 2005; Bustos-Pérez, 2025; Hiscock & Attenbrow, 2003; Hiscock & Clarkson, 2009; Hiscock & Tabrett, 2010; Kuhn, 1990; Marwick, 2008; Morales & Vergès, 2014; Morales et al., 2015). These quantitative methods can sometimes estimate the hypothetical initial volume of the tool blank, allowing us to predict differences in the variation of the tool along the two scaling directions (allometric versus isometric reduction trajectories (Iovita, 2009, 2010, 2011). Our approach examines the waste material resulting from this reduction, a by-product of the maintenance techniques.

Our approach stems from the principle of the *chaîne opératoire,* that each artefact can be placed within the space and the time of the flintknapping activity, (Soressi & Geneste, 2011, p. 337). In this sense, we aim to demonstrate that incorporating the module flake categories into the reduction sequence can significantly aid in unpacking the complexities of decision-making regarding tool use and maintenance. We believe that we can now better test retouch intensity in lithic assemblages by using the quantitative approach based on tools, which is a static approach, and linking them to the frequencies of categories of module flakes that are representative of the dynamic behaviour of retouching, reshaping, and rejuvenating, giving us a maintenance index. Furthermore, by binding the classification of raw material units to the module flake categories, we can attempt to quantify raw material curation in terms of the degree of extracted utility per raw material relative to the distance from its source (e.g., Nora et al., 2025a; Frahm et al., 2025; Nora et al., in prep, Kalavan 2 paper).

Our analysis set out with explicit expectations: (a) that dorsal scar counts would increase from early (M0-M1-M2) to later modules (M3-M4), reflecting cumulative edge modification; (b) that mass would also increase across modules, providing an independent proxy for material investment in maintenance; and (c) that the most advanced stage (M4) would diverge from earlier categories, representing rejuvenation events and marking a tipping point in tool use-life. The results presented here support the interpretive value of the module flake classification as a tool for understanding patterns of maintenance strategies. Non-parametric tests conducted across the three assemblages at Kalavan 2 T1, Kalavan 2 T2, and Ararat-1 Cave demonstrate statistically significant differences in dorsal scar count, approximate area, and mass across module categories (M0–M4). The Kruskal–Wallis tests revealed overall variation, while Mann–Whitney U tests with Bonferroni correction confirmed that most contrasts, particularly between early (M0–M1) and later (M3–M4) stages, are significant. Dorsal scar count increases consistently with module level, showing a clear accumulation of retouch. The approximate area also tends to increase in higher modules, although this pattern is more variable between sites. This suggests that while scar accumulation reflects a continuous trajectory of edge modification, increases in flake size are likely linked to intensifying maintenance and are more variable and context dependent. The plateau in scar count observed between M3 and M4 in some cases may indicate a saturation point on the edge, or a final phase in a tool’s use-life before discard, or reshaping and transport, as evident by the “ghost tools”, tools that are no longer present in the archaeological record.

Among the three measured variables, mass stands out as the most independent and interpretable. Unlike scar count, which is partially involved in the classification criteria, mass is not part of the definitional framework and thus provides more external data on the classification system. Mass values consistently increase across modules, reflecting the cumulative material removed through maintenance activities. This makes mass a valuable proxy for estimating the intensity of edge modification. More importantly, these values offer a foundation for future modelling of mass loss, both in relation to raw material units and to edge dimensions. With appropriate geometric clustering and volumetric estimates, it may be possible to predict the average amount of mass removed during specific phases of maintenance or to calculate how much material is typically needed to reshape a given edge length. This potential extends the interpretive knowledge of the module framework beyond classification alone, contributing to broader questions of tool use-life, raw material economy, and provisioning strategies.

When dealing with provisioning strategies, module classification can distinguish the initial approach towards the blank. M0 (primary edge modification) is conceptually distinct from the other modules, as shown through the Mann–Whitney U tests. The absence or low frequencies of M0 flakes among the module flake assemblage can suggest that the initial edge modification occurred off-site. This could indicate a curated provisioning strategy where pre-modified blanks or partially retouched tools were transported to the locality. In contrast, high frequencies of M0 with the parent scar preserved guide us to the perspective of unmodified blanks at the locale, which can be represented by on-site blank production (when compared to raw material units, core presence, unmodified blanks, and core trimming elements) or the transportation of blanks into the locality. The presence of M1, M2, and M3 indicates that blanks are at a stage of secondary edge modification, implying the material either entered the site already retouched or was retouched on-site. For example, in Ararat-1 Cave, the high frequency of M2 and M3 made on Arteni obsidian sources suggests that these pieces arrived at the site in a partially worked state, and a more advanced retouching event (51–95% of edge modification) took place on-spot (Nora et al., 2025a). This segmented representation of the module flakes could indicate that the specific lithic raw materials became more important over time, potentially reflecting shifts in raw material availability and exploitation. The high frequency of M3 suggests raw material economisation with a significant focus on these sources during more advanced stages of edge maintenance. The interpretation of Module 4 flakes indicates a tipping point in tool maintenance, where retouching leads to reshaping/rejuvenation of the edge (Fig. 8). The frequent occurrence of M4 flakes suggests that tools were continuously maintained on-site, either for further use or transport.

(Shott, 1989, p. 24, 1996) suggested that curation is a concept directly related to the tool's initial maximum utility and how much of that utility is realised before it is discarded. Following this perspective, we can attempt to measure curation through the module flake categories, taking into consideration the RMU as our additional analytical unit (Odell, 2004, pp. 93–95; Roebroeks et al., 1988). The pattern we observed either from the raw material strategies and the maintenance strategies seen by the module flakes is consistent with curation strategies where the goal is to prolong the life of the tools through repeated rejuvenation, ensuring that high-quality materials like obsidian are utilised to their fullest potential (Nora et al., 2025a; Odell 1996). The single representation of M4 for the most distant raw materials, such as Chikiani obsidian in Kalavan 2 T1 (Malinsky-Buller et al., 2021), suggests that some tools entered the site and were taken away after reshaping/rejuvenation. This evidence of “ghost tools” is represented only by the byproducts that indicate maintenance activity at the locale. This pattern of reshaping tools to create new, sharp edges and then removing them from the site is indicative of tool provisioning for continued use elsewhere, supporting a mobile strategy where tools were maintained at key locations and then transported to other areas of activity, sensu (Bousman, 1993).

**Fig. 18:**
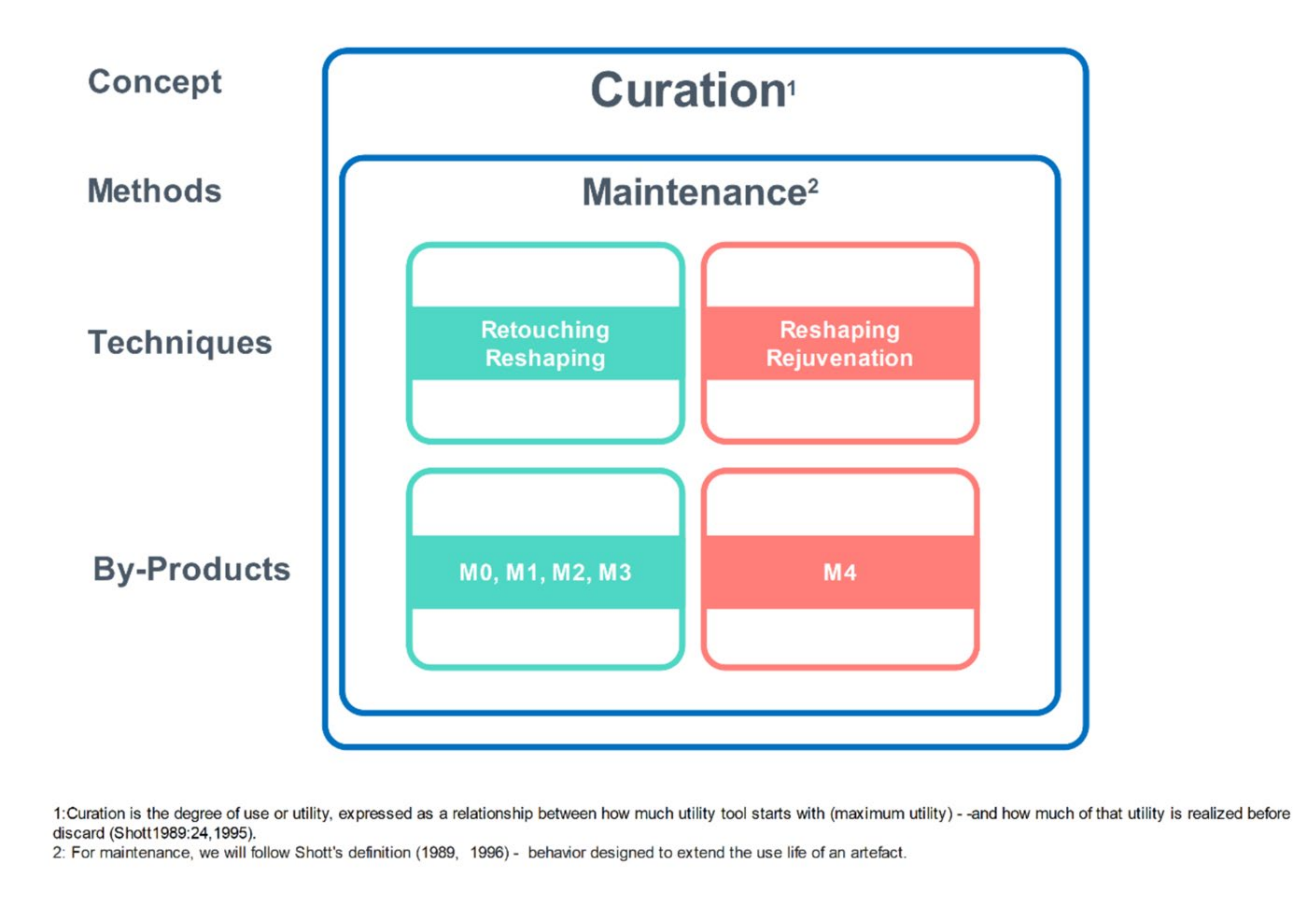
Conceptual model linking edge modification modules to tool curation and maintenance strategies. Modules 0–3 reflect retouching and reshaping behaviours, while Module 4 is associated with reshaping and rejuvenation. Definitions follow Shott (1989, 1996), with curation as utility over time and maintenance as behaviours extending tool use-life

The categorisation we put forward here aims to encompass the various typologies used in relation to the concept of curation and, consequently, the maintenance of lithic tools (see Fig. 18), emphasising that their behavioural signature is more significant than typological attribution (see discussion in (Bar-Yosef & Van Peer, 2009) for the use of technological traits vs. typological). The suggested categorisation reflects a behaviourally meaningful model that corresponds to measurable changes in artefact form. For example, a single M4 flake removal would create a clean edge, contrasting with a wavy edge shaped by a succession of M0/M1/M2/M3 removals. Thus, interpreting the module flakes within a technological perspective provides a nuanced reconstruction of past decision-making. The suggested categorisation can be divided into the first edge modification (M0), the retouching/reshaping (M1/M2/M3), and finally, the reshaping/rejuvenation phase (M4) (Fig. 18). Those techniques, although conceptually closer, differ in their gestures and intentionality (Forestier, 1993; Inizan et al., 1995). The different module flakes enable us to reconstruct at what stage blanks were transported to a given locality, either as unretouched blanks or as retouched items, and if the latter, to what degree. The progressive increase in dorsal scar numbers and the selective increase in artefact size confirm that the module framework captures distinct categories within situational decision-making during the life history of lithic tools. This reinforces the model's utility for comparative and inferential studies of lithic maintenance strategies within archaeological sites.

### Tackling the Limitations of Module Flake Categories

The scope of this study is to bring microdebitage to the forefront of lithic analysis, arguing that these byproducts convey meaningful behavioural signals. The proposed five-tier module categories (M0–M4) represent a critical step toward decoding maintenance intensity, tool mobility, and provisioning strategies. However, like any heuristic model, this framework has both strengths and limitations. Recognising these constraints is essential for refining our interpretation of lithic reduction and developing robust analytical tools for future research. One of the challenges lies in cross-comparing module flakes with flakes produced from core preparation, blank production, and platform preparation. These small-sized artefacts, in theory, given their morphology, can be mistaken for some ground with module flakes. Another critical limitation that can be pointed out is the absence of expected proportions for each module category during maintenance activities. In this paper, invasiveness was estimated through visual assessment of dorsal scar coverage, layering, and count. While faster, this method can introduce a sense of subjectivity among peers and limit replicability.

The experimental study by Kot et al. (2024) is a reminder of the inherent information loss embedded in lithic reduction, an issue that has been explored since Villa (1982), Kimura (1999, 2002, 2006), Braun et al. (2005), and Braun et al. (2006). Kot et al., (2024) quantitative refitting analysis demonstrates that cores retain only around 40% of the removals and approximately 14% of the sequential relationships between them, leading the authors to note that only the very last removal is certain. This observation, which refers to the difficulty of reconstructing the successive stages of a tool’s use-life, directly frames one of our analytical premises: rather than attempting to follow each removal event, we delimit the first observable modification that happens in the edge (M0) and the rejuvenation stage (M4) as the end of a behavioural continuum. At the same time, Kot et al. report a strong positive correlation between the true number of removals and the number of visible scars (ρ = 0.88, p < 0.0001), showing that areas knapped with higher intensity still bear proportionally more scars. This result supports the use of scar counts as relative, not absolute, indicators of reduction intensity, as we do in our module analysis. Thus, while acknowledging that chronological detail is lost, we maintain that patterned variation in scar frequency and invasiveness retains meaningful behavioural information. To address these limitations, we are currently developing an experimental framework designed to quantify the production, geometry, and improve the behavioural context of microdebitage flakes at different stages of the reduction process. The suggested experimental program will take into consideration the issue of flake recovery rate, sensu (Kot et al., 2024), demonstrating that flintknapping is an inherently lost process in which much of the procedural sequence is not preserved in individual debitage. In this framework, flakes are generated through controlled sequences of tool production, use, and maintenance activities across different lithic raw materials and contact materials. We aim to 3D scan each flake and tool as part of the sequential experiment, similar to that of (Nora et al., 2025b), enabling us to extract high-resolution geometric attributes, including platform angle, platform depth, dorsal scar complexity, and surface-to-volume ratio. Our module flake classification does not aim to recover precise knapping sequences. Instead, it identifies cumulative scar patterns and morphological changes that reflect broader behavioural phases of edge modification. The classification operates at the population level, capturing recurrent traits linked to maintenance intensity. This experimental approach will provide an empirically grounded baseline for the external validation of module frequencies (Eren et al., 2016) reduce subjectivity in classification and enhance the resolution of behavioural inferences derived from microdebitage, specifically module flakes, as discussed in this paper.

## Conclusions

Applying our proposed module flake categorisation (M0–M4) to the late Middle Palaeolithic artefact assemblages of Kalavan 2 T1 and T2 and Ararat-1 Cave offers new insights into the fluidity of the technological processes underlying tool maintenance. The systematic classification of byproducts, regardless of size, enabled us to reconstruct detailed edge modification sequences and tool use-life. The identification of M0 indicates the first approach to an unretouched blank, while a high frequency of M1, M2, and M3 module flakes revealed extensive initial edge modifications and ongoing retouching activities. These patterns reflect adaptive strategies for tool maintenance, which can also be used to explore retouch intensity. M4 flakes, representing advanced stages of tool use, suggest prolonged use and retouch intensity of tools that lead to a blunt or unstructured edge, as noted by Frison (1968). This dynamic module category goes beyond a linear sequence by considering edge modifications, overlapping, and an iterative nature. Rather than a linear progression of retouch, we offer a “snake and ladder” approach, emphasising the fluidity of decision-making (Fig. 8). The analysis underscores the critical role of large and small waste flakes in understanding past technological behaviours, stressing that byproducts are integral to a holistic reconstruction of prehistoric tool use-life, rather than merely waste material. By applying this novel framework, we have expanded the interpretative potential of lithic assemblages, shedding light on the often-overlooked smaller fractions. Those pieces hold significant information about the intensity and nature of tool use, tool maintenance practices, and mobility strategies. This approach offers a more nuanced understanding of tool curation, contributing to broader discussions on Palaeolithic technological organisation and putting the decision-making processes of tool maintenance at the focal point of inquiry.

## Data Availability

The datasets generated during and/or analysed during the current study are available at: [https://github.com/Nora-Arch/Moduleflakescategories] (https:/github.com/Nora-Arch/Moduleflakescategories).
